# Mechanochromic, Structurally Colored, and Edible Hydrogels Prepared from Hydroxypropyl Cellulose and Gelatin

**DOI:** 10.1002/adma.202102112

**Published:** 2021-07-29

**Authors:** Charles H. Barty‐King, Chun Lam Clement Chan, Richard M. Parker, Mélanie M. Bay, Roberto Vadrucci, Michael De Volder, Silvia Vignolini

**Affiliations:** ^1^ Department of Engineering University of Cambridge 17 Charles Babbage Road Cambridge CB3 0FS UK; ^2^ Yusuf Hamied Department of Chemistry University of Cambridge Lensfield Road Cambridge CB2 1EW UK

**Keywords:** cholesteric liquid crystals, edible hydrogels, hydroxypropyl cellulose, mechanochromic materials, photonic hydrogels

## Abstract

Hydroxypropyl cellulose (HPC) is an edible, cost‐effective and widely used derivative of cellulose. Under lyotropic conditions in water, HPC forms a photonic, liquid crystalline mesophase with an exceptional mechanochromic response. However, due to insufficient physical cross‐linking photonic HPC can flow freely as a viscous liquid, preventing the exploitation of this mechanochromic material in the absence of any external encapsulation or structural confinement. Here this challenge is addressed by mixing HPC and gelatin in water to form a self‐supporting, viscoelastic, and edible supramolecular photonic hydrogel. It is demonstrated that the structural coloration, mechanochromism and non‐Newtonian shear‐thinning behavior of the lyotropic HPC solutions can all be retained into the gel state. Moreover, the rigidity of the HPC‐gel provides a 69% shorter mechanochromic relaxation time back to its initial color when compared to the liquid HPC–water only system, broadening the dynamic color range of HPC by approximately 2.5× in response to a compressive pressure. Finally, the ability to formulate the HPC‐gels in a scalable fashion from only water and “food‐grade” constituents unlocks a wide range of potential applications, from response‑tunable mechanochromic materials and colorant‐free food decoration, to short‐term sensors in, for example, biodegradable “smart labels” for food packaging.

## Graphical abstract

A self‐supporting and structurally colored hydrogel is produced by combining hydroxypropyl cellulose (HPC) and gelatin in a scalable formulation process. By retaining the lyotropic, liquid crystalline behavior of the HPC into the gelled state, a spectrum of dynamic colors is produced. Rheological studies confirm a desirable non‐Newtonian shear‐thinning behavior and an increased stiffness, which result in a much quicker mechanochromic relaxation time.

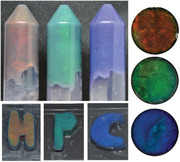

## 1 Introduction

Hydroxypropyl cellulose (HPC) is a widely utilized, water soluble derivative of cellulose. Its rheological properties and biocompatibility have led to extensive use in the medical, pharmaceutical and food industries, as an eye‐treatment,^[^ ^1^ ^]^ bulking and drug‐release agent,^[^ ^2^, ^3^, ^4^ ^]^ and as a thickener and stabilizer,^[^ ^5^, ^6^ ^]^ to mention only a few. Despite its widespread use and seminal photonic studies in the 1970s and 1980s, the development of HPC‐based photonic applications has been slow.^[^ ^7^, ^8^, ^9^, ^10^, ^11^, ^12^, ^13^ ^]^ Recently however, a growing interest in sustainable raw materials and their application has seen a shift in photonic HPC research toward practical applications^[^ ^14^, ^15^, ^16^, ^17^, ^18^, ^19^, ^20^, ^21^, ^22^ ^]^ and large‐area processing.^[^ ^17^, ^18^ ^]^

**Figure 1:**
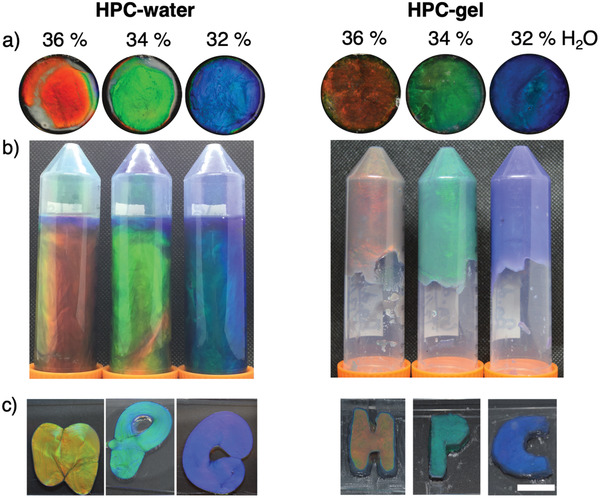
Red, green, and blue samples of HPC‐water (left) and HPC‐gel (right), corresponding to 36, 34, and 32 wt% water, respectively; all contain 0.005 wt% nigrosine. a) Samples contained within 6 mm thick rubber O‐rings, sealed between glass slides with epoxy glue. Circles are 20 mm in diameter. b) Samples in Falcon tubes (50 mL) placed upside down for 48 h. c) Free‐standing samples placed between two glass slides and left at rest for at least 1 min under gravity. The white scale bar is 1 cm.

Aqueous HPC is known to self‐assemble into a cholesteric liquid crystal under ambient conditions when the water content falls below around 45 wt%.^[^ ^8^, ^23^, ^24^ ^]^ The cholesteric phase has a periodic, helicoidal nanostructure, defined by a physical distance called the pitch, *p*, which decreases as the water content is reduced.^[^ ^17^, ^23^ ^]^ When *p* is on the length scale of the visible spectrum, incident light is selectively reflected in a similar fashion to Bragg‐reflection and the HPC mesophase displays a vivid metallic coloration (Figure **1**).^[^ ^25^ ^]^ The observed color is predominantly determined by the type of HPC used and the solvent concentration.^[^ ^9^, ^17^, ^26^ ^]^ However, the color can still be dynamically controlled post‐formulation by actively manipulating the cholesteric pitch. For example, applying a macroscopic pressure will compress the cholesteric phase, reducing *p* at the point of contact, and visually result in a localized and reversible blueshift,^[^ ^17^ ^]^ termed mechanochromism.

A mechanochromic response, combined with large‐scale production, extensive commercial use and certification for human consumption,^[^ ^27^ ^]^ affords HPC great potential in biocompatible and cost‐effective sensing applications.^[^ ^17^, ^18^, ^28^, ^29^, ^30^ ^]^ However, while recent studies have been successful at converting the HPC mesophase into a fully solid‐state photonic structure, for example via chemical cross‐linking or the further functionalization of HPC side‐chains,^[^ ^11^, ^22^, ^31^ ^]^ this has come with a loss of the dynamic color response. As such, HPC mechanochromism has only been reported in liquid formulations to date. In this study, we demonstrate a mechanochromic HPC‐gel using only cost‐effective, biocompatible, and widely available raw materials. We show that the HPC‐gel is moldable as a continuous unsupported solid, while retaining a shear‐thinning non‐Newtonian response that is preferable for liquid processing. Finally, we show that the mechanochromic relaxation time of the photonic HPC‐gel is significantly enhanced over the equivalent HPC‐water mesophase, making HPC‐gels interesting for short‐term visual sensing applications where biocompatibility or biodegradability are essential.

## 2 Results and Discussion

To improve the mechanical response of the colored HPC mesophase, gelatin was chosen as the supramolecular gelling agent due to its edibility, water‐soluble nature, and commercial ubiquity. By mixing HPC, gelatin and water in a planetary centrifugal mixer, a photonic and viscoelastic HPC‐gel was obtained, as shown in Figure 1 (full formulation details are provided in the Experimental section). After homogenous mixing, the materials dissolution was enhanced using tepid water baths, the air bubbles removed via centrifugation, and the hydrogel set by refrigeration. Nigrosine, a water‐soluble black dye, was also included as a broadband absorber to enhance the saturation of the reflected color (Figure S1b, Supporting Information). Note that nigrosine can be replaced with, for example, carbon black, to allow a fully edible, photonic HPC‐gel to be formulated (Figure S1a, Supporting Information). However, the particulate nature of carbon black disrupts effective spectroscopic data acquisition and as such nigrosine was predominantly used in this study to allow quantitative optical analysis.

**Figure 2:**
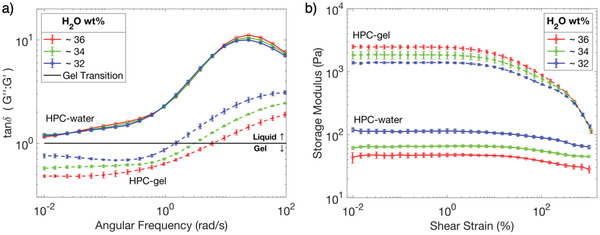
Rheological sweep profiles of samples of HPC‐water (solid lines) and HPC‐gel (dashed lines) with decreasing water content, recorded at 20 °C. An average of three runs is shown for each plot and the standard deviation per point is given by the error bars. a) Rheological frequency sweep profiles decreasing from 100 to 0.1 rad s^−1^ angular frequency, at constant 0.01% applied strain. The plot shows the loss factor (tanδ = *G*ʹʹ/*G*ʹ), the ratio between the viscous (*G*ʹʹ) and elastic (*G*ʹ) contributions, where tanδ > 1 = viscoelastic liquid, and tanδ < 1 = viscoelastic solid. The horizontal black line denotates the gel‐transition point (tanδ = 1). b) Rheological amplitude sweep profiles increasing from 0.01 to 1000% applied strain, showing the storage modulus, *G*ʹ, at constant angular frequency of 10 rad s^−1^. For clarity the loss modulus, *G*ʹʹ, is not shown.

The aqueous HPC mesophase and corresponding HPC–gelatin hydrogel are compared in Figure 1. Both systems display strong structural coloration, indicating that the self‐assembly of HPC into a photonic, cholesteric liquid crystal is not disrupted by the presence of gelatin (Figure 1a). A significant macroscopic rigidity is however introduced into the HPC‐gels, preventing the flow of material under its own weight (Figure 1b), and allowing self‐supporting shape retention that is not observed in the viscous, yet still liquid, HPC‐water analogue (Figure 1c). The stiffness of the HPC‐gel is attributed to the introduction of gelatin leading to increased elastic contributions,^[^ ^32^ ^]^ as confirmed by rheological analysis (**Figure** **2**). Additionally, the water‐dependent lyotropic nature of HPC remains the determinant factor for the observed color,^[^ ^9^ ^]^ with a similar water content in the two systems resulting in comparable coloration (despite a decrease in HPC content as gelatin is introduced). However, a slight increase in turbidity was observed in the HPC‐gel, possibly due to a degree of immiscibility between the HPC and gelatin polymers,^[^ ^33^ ^]^ leading to a more matte visual appearance.

The rheological frequency sweeps, presented in Figure 2a, show the time‐dependent behavior of the two HPC systems as a function of descending angular frequency from 100 to 0.01 rad s^−1^. The gel‐transition point, where the loss factor (tanδ = 1),^[^ ^34^ ^]^ is given as a horizontal black line. The experimental conditions were determined by preceding rheological amplitude sweeps to find the materials linear viscoelastic region, as shown in Figure 2b. Furthermore, by inducing a high frequency shear and reducing its frequency towards a rest state, the descending frequency sweep allows for investigation of the internal structure of the material and its recovery towards rest.^[^ ^34^ ^]^ As observed in Figure 2a, the HPC‐water mesophase (solid line) exhibits characteristic viscoelastic‐liquid‐like behavior over all frequencies,^[^ ^35^, ^36^, ^37^ ^]^ though comes close to a gel transition point toward rest. In contrast, the HPC–gelatin hydrogel (dashed line) recovers from a liquid‐like state, induced by the initial higher frequencies of the measurement, to that of a viscoelastic solid‐like state as the angular frequency is reduced. A gel‐transition point occurs between 1 and 6 rad s^−1^. At rest, the elastic contributions of the HPC‐gel dominate over its viscous component and the sample forms a hydrogel (i.e., tanδ < 1). This allows the material to resist macroscopic flow and retain a prescribed shape, as exemplified respectively in Figure 1b,c. However, a liquid‐like behavior was still observed at angular frequencies exceeding the gel transition point, indicative of a non‐Newtonian response that is beneficial for physical processing.^[^ ^34^ ^]^ Indeed, HPC‐water is known to exhibit considerable non‐Newtonian, shear‐thinning pseudoplasticity even at low rates of shear,^[^ ^36^, ^37^ ^]^ as validated in Supporting Figure S2. Such behaviour has also been observed in other, non‐photonic, HPC hydrogels that display shear‐thinning rheological behavior at very low HPC concentrations.^[^ ^36^ ^]^ Similarly, a strong shear‐thinning non‐Newtonian response is observed in the HPC–gelatin hydrogel (Figure S2, Supporting Information) to the extent that a liquid–gel transition point occurs at increased rates of shear and allows the material to flow as a liquid (Figure 2a), despite the order of magnitude increase in the elastic modulus, *Gʹ*, compared to HPC‐water (Figure 2b). After the shear is removed, HPC‐gel resolidifies back into a hydrogel as the gel transition point is crossed again toward rest. The characteristic shear‐thinning properties of HPC therefore remain largely unperturbed by the presence of gelatin, similar to the photonic characteristics reported in Figure 1. Finally, it is interesting to note that the rheological amplitude sweeps, presented in Figure 2b, indicate that opposite trends in *Gʹ* occur for the two systems as the water content is reduced. In HPC‐water, *Gʹ* is dictated by the HPC content; the other constituent being water. The progressive replacement of water with HPC, therefore, results in a contraction of the cholesteric pitch (and corresponding blueshift in the reflected color),^[^ ^9^ ^]^ and an increase to the elastic modulus *Gʹ* (Figure 2b). However, in the HPC‐gel, a contraction of the cholesteric pitch (and therefore a blue‐shift in the color) has the opposite trend to *Gʹ*. This effect arises because gelatin, not HPC, is now dictating the storage modulus of the HPC‐gel system. The progressive replacement of water with HPC therefore results in less water available for dissolution of the gelatin and a decrease in *Gʹ* is observed as the formulation is blueshifted.

To address how HPC‐gel retains its photonic properties into the viscoelastic gel state, the rheological behavior must first be understood. Intermolecular association, penetration and entanglement, and the disruption thereof, commonly dictate the plastic behavior of polymer solutions,^[^ ^34^, ^38^ ^]^ with HPC‐water, as well as some gelatin and gelatin‐blends, known to exhibit shear‐thinning behavior independently.^[^ ^36^, ^37^, ^39^, ^40^ ^]^ We postulate that the mechanism for the observed shear‐thinning behavior of the HPC‐gels (from a gel at rest, to a liquid under high rates of shear, to a gelled state again toward rest) is the result of supramolecular physical cross‐linking giving rise to a thermoplastic interpenetrating polymer network, and its subsequent disruption.^[^ ^41^ ^]^ On formulation, a continuous entangled superstructure of gelatin is formed that spans the whole sample and is interspersed with discrete HPC photonic domains. These regions of predominantly HPC mesophase dominate the photonic characteristics (Figure 1a), while the macroscopic stiffness is provided by the hydrogelation of the gelatin (Figure 1b,c). However, at increasing rates of shear, a disentanglement occurs that disrupts this sample‐spanning gelatin superstructure and decreases the flow resistance, reducing the viscosity,^[^ ^34^ ^]^ and giving rise to the characteristic shear‐thinning behavior (Figure S2, Supporting Information). As the shear is subsequently reduced towards rest, a dynamic re‐entanglement and relaxation back to the original gel state occurs, allowing the continuous gelatin superstructure to reform and provide macroscopic stiffness once more (Figure 2a).

**Figure 3:**
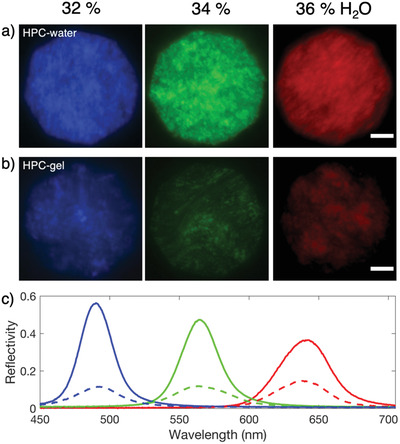
a,b) Bright‐field microscopy images comparing the optical appearances of HPC‐water (a) and HPC‐gel (b) for increasing water content (wt%) and imaged through a right‐handed circular polarized filter. The white scale bars are 50 µm. c) Corresponding micro‐spectroscopy of the HPC‐water (solid lines) and HPC‐gel (dashed lines). Each curve is an average of three spectra per sample and acquired at different locations.

To quantitatively characterize the lyotropic photonic behavior of HPC‐water (**Figure** **3**a) and HPC‐gel (Figure 3b), polarized optical microscopy and micro‐spectroscopy was performed (Figure 3). All spectra reported in Figure 3c were captured in bright‐field illumination, with the reflected light passing through either right‐hand circular polarized (RCP) or left‐hand circular polarized (LCP) filters. It was found that all samples reflect predominantly RCP light (Figure 3), with only a weak signal observed to be LCP (Figure S3, Supporting Information), attributed to the depolarization from scattering and defects within the cholesteric mesophase.^[^ ^13^ ^]^ We also observe that the measured peak wavelength (and correspondingly the cholesteric pitch) is comparable between samples of the same water content, albeit with a reduction in the reflected intensity of the HPC‐gel under bright‐field illumination. These observations are attributed to the microphase separation of discrete HPC regions within a continuous gelatin network, which results in increased disorder and scattering between individual cholesteric domains. Furthermore, the reduction in intensity (Figure 3c) could also be attributed to a reduction in the size of these discrete cholesteric domains, as compared to the continuous mesophase present in HPC‐water. Similarly, comparable wavelengths and a reduced intensity between the two systems were observed in spectroscopic goniometer plots, with an angular‐dependence to the reflected color confirming that photonic HPC‐gel is iridescent (Figure S4, Supporting Information). Overall, the lyotropic photonic behavior of the HPC mesophase is retained into the gel state, without a significant shift in the reflected wavelength from the presence of an extended gelatin network.

**Figure 4:**
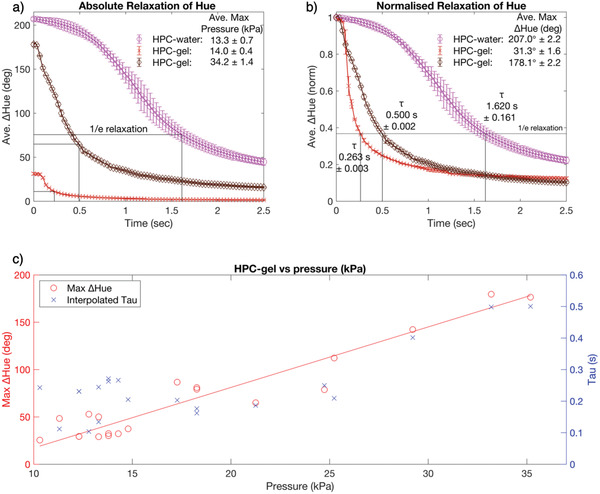
a,b) Interpolated mechanochromic recovery of red samples of HPC‐water (purple circles) and HPC‐gel (red crosses) under a comparable compression, and HPC‐gel (brown diamonds) under a comparable color change to HPC‐water, plotted in terms of absolute change in hue (ΔHue) (a) and self‐normalized to the maximum hue recorded (b). The time constant, τ, indicates the time taken for all pixels within the region of interest to decay to 1/*e* ≈ 36.8% of their original value. Each plot is an average of two comparable experiments. c) A color‐pressure plot showing the maximum ΔHue and corresponding τ recorded for HPC‐gel upon compressions of different magnitude.

Thermoplastic interpenetrating polymer networks typically retain the individual characteristics of their constituent polymers into the new mixture via an entanglement or penetration of one polymer network into the other, providing an improved, complementary performance.^[^ ^38^ ^]^ In the case of the HPC‐gel, its shear‐thinning rheological response (Figure S2, Supporting Information) and relaxation toward rest (Figure 2a) enables the interspersed photonic domains of HPC to respond dynamically within the polymer skeleton of gelatin. The notable mechanochromism of the HPC mesophase,^[^ ^17^, ^18^ ^]^ therefore, is retained into a self‐supporting gel state. To quantify how the introduction of an extended gelatin network impacts this mechanochromic behavior, the color responsiveness of the two systems was compared, as shown in **Figure** **4**. A water concentration corresponding to an overall red coloration was chosen for both systems to enable compression‐induced blueshifts through the full visible spectrum. The HPC‐gel and HPC‐water formulations were cast into 5.5 cm diameter, 1 cm deep Petri dishes and a finger pressure exerted and released. Any resultant HPC color change was recorded in real time using a camera. Within the region of interest (ROI), RGB (red, green, blue) pixel values for each frame were then converted to the HSL (hue, saturation, lightness) color space,^[^ ^18^ ^]^ and the hue values averaged over two independent but comparable “finger presses.” The framerate was then used to plot the change in hue (ΔHue) as a function of time (Figure 4a), as well as normalized to the maximum hue change recorded (Figure 4b). The applied pressure was logged concurrently using a force sensor placed at the ROI. By approximating the time response to an exponential decay, a mechanochromic time constant, τ, could be calculated for each sample to represent the duration required for the hue to fall back to 1/e of its initial value.

By first considering the application of a comparable compressive pressure (approximately 14 kPa, purple circles vs red crosses), it was observed that HPC‐water underwent a large mechanochromic response from red‐to‐blue (ΔHue = 207°), with a mechanochromic relaxation time back to its initial state of τ = 1.62 s. In contrast, applying this pressure to the HPC‐gel resulted in a much smaller color shift (ΔHue = 31°, i.e. 85% less) and a reduced mechanochromic relaxation time of τ = 0.26 s (i.e. 84% shorter). These reductions are attributed to the increased stiffness of the HPC‐gel and thus its resistance to deformation, preventing the applied pressure from sufficiently compressing the sample to induce a full spectrum color shift. However, when a larger pressure was applied to the HPC‐gel (34 kPa, brown diamonds), a mechanochromic response through the full visible range was now observed (ΔHue = 178°), while still retaining an improved mechanochromic relaxation time (τ = 0.50 s, i.e. 69% shorter). These results confirm that the mechanochromic relaxation responsivity of the HPC‐gel system is significantly enhanced when compared to HPC‑water.

The mechanochromic response of the HPC‐gel to a series of different applied pressures is reported in Figure 4c. Importantly, this plot confirms that the mechanochromic response is linear across the pressure regime of interest, with the mechanochromic sensitivity calculated from the trendline to be 6.4 (± 0.4)° kPa^−1^. Upon an application of pressure to the HPC‐gel, regions of predominantly HPC will experience a compression and consequently blueshift, yet, unlike HPC‐water, they cannot inelastically flow due to the localized confinement by the surrounding extended HPC–gelatin network. Furthermore, the macroscopic stiffness of this network resists deformation, providing a viscoelasticity that shortens the mechanochromic relaxation time. As such, the change in hue is reduced and the mechanochromic relaxation time is shortened compared to the analogous HPC‐water mesophase. This has the effect of broadening the pressure range over which a full‐spectrum color mechanochromic response can be observed, with a factor of approximately 2.5 measured for the reported HPC‐gel (i.e., 0–14 vs 0–35 kPa). This represents a key improvement on the narrow dynamic color‐pressure range typically reported for HPC sensors.^[^ ^18^ ^]^ Furthermore, suppression of the lateral flow of HPC, which dominates in HPC‐water,^[^ ^17^, ^18^ ^]^ could potentially allow for greater spatial resolution. Finally, where a given application requires a specific sensitivity, further optimization of the formulation by, for example, varying the gelatin content, will allow for fine‐tuning of the stiffness and consequently the mechanochromic response.

## 3 Conclusions

Widely available and biocompatible building blocks have been combined in a readily scalable formulation process (planetary centrifugal mixing) to produce a structurally colored HPC–gelatin hydrogel that can be both molded as a solid yet processed as a liquid. The desirable lyotropic‐dependence of the photonic, liquid crystalline HPC mesophase is retained into the gel state. Furthermore, the HPC‐gel retains the characteristic mechanochromism of HPC into a self‐supporting viscoelastic solid structure, but with a much shorter mechanochromic relaxation time upon comparable full visible spectrum color shifts. This offers a practical broadening of the dynamic pressure range that can produce a visible mechanochromic response. These effects are attributed to the increased elasticity upon introduction of just 7.0 wt% porcine gelatin to the HPC mesophase. A significant shear‐thinning pseudoplasticity is also retained that enables a gel‐to‐liquid transition at increased rates of shear, relaxing back to a self‐supporting gel state once the shear is reduced. The produced HPC‐gels are therefore shown to be versatile materials for rapid commercial development, with their shear‐thinning behavior allowing for well‐established industrial processing techniques, such as extrusion,^[^ ^35^ ^]^ casting,^[^ ^11^ ^]^ or high‐speed rotary‐spinning,^[^ ^42^ ^]^ in a large‐scale production setting. Finally, as this system could be fully realized at scale with commercially available edible constituents, a wide range of potential cost‐effective mechanochromic materials are unlocked, from colorant‐free food decoration, to short‐term sensors in, for example, biodegradable “smart labels” for food packaging.

## 4 Experimental Section

### Materials

Ultrapure Type 1 water was used for all samples (Merck Millipore, Synergy System). Dry hydroxypropyl cellulose was supplied by NISSO Chemical Europe (HPC SSL SFP, food grade, *M*_w_ 40 000 g mol^−1^ as reported by manufacturer). Gelatin powder was supplied by Sigma‐Aldrich (porcine‐derived, type A, bloom strength 300). The black contrast materials used were water soluble nigrosine dye (Alfa Aesar, A18147) and powdered carbon black (Alfa Aesar, acetylene, 50% compressed, 99.9+%). Carbon black is approved for use as the European food additive (E152), allowing for edible and high contrast photonic HPC formulations (Figure 1, Supporting Information). However, although both black materials provide visually similar contrast enhancement, the particulate nature of carbon black disrupts effective microspectroscopic data acquisition under the microscope and as such nonedible nigrosine was preferred for quantitative analysis. All materials were used as received from the supplier.

### Sample Formulation

For a given sample, the relative proportion of constituents were measured by their mass ratio (wt%) to a total of 55 g per sample and were accurate at the time of weighing (not measured postformulation). Red, green and blue samples contained 36, 34 and 32 wt% water, respectively, as well as either 0.05 wt% of carbon black (Figure S1 only, Supporting Information) or 0.005 wt% nigrosine dye (all other samples). For the HPC‐gels, 7.0 wt% of gelatin powder was included in all samples. The remaining mass comprised sieved HPC powder. Materials were directly combined in order, either: water, nigrosine, HPC, gelatin, or: water, HPC, carbon black, gelatin, and mixed to homogeneity using a planetary centrifugal mixer (ThinkyMixer ARE‐250). Three sequential mixing steps were used in a continuous manner: i) a wetting step (1600 rpm for 2 min), ii) a soak step (0 rpm of 2 min), and iii) a homogenous mixing step (1800 rpm for 2 min). After mixing, samples were poured into falcon tubes (50 mL), whereupon they were sealed and placed into a water bath (33 °C) for 1.5–2.0 h to facilitate dissolution. The samples were then centrifuged (Heraeus Multifuge X1R, Thermo Scientific, 33 °C, RCF 11 617 × *g*, 45 min) to remove trapped air bubbles before being placed back into the water bath for a further 1.5–2 h to ensure complete dissolution. All samples were then stored at 4 °C for a minimum of 48 h to promote gelation. Before preparation for data acquisition (see below), samples were removed from the fridge and maintained at 20 °C to equilibrate for a minimum of 6 h, though typically much longer (12 h or more).

### Rheology

A rheometer (TA Instruments, Discovery HR‐2) with a 20 mm parallel plate top geometry was used with a 1 mm gap size at 20 °C. Oscillatory tests were used for all measurements, except the rotational flow sweeps in Figure S2 (Supporting Information). An empty solvent trap was used to fully enclose samples during data acquisition so that water loss to ambient air flow could be minimized. Each formulation was measured three times and averaged, with a fresh sample used for each measurement. Frequency sweeps: The angular frequency was ramped down from 100 to 0.01 rad s^−1^ at a constant applied strain of 0.01%, informed by the linear viscoelastic region, LVR, obtained from amplitude sweeps. A descending ramp was used to mimic the time‐dependent relaxation of the material from high to low rates of strain. Amplitude sweeps: An applied strain was ramped from 0.01 to 1000% at a constant angular frequency of 10 rad s^−1^. The LVR from the response curves informed the parameters for the frequency sweeps. Viscosity–shear rate profiles: The shear rate was ramped from 0.01 to 100 s^−1^, while the viscosity was recorded.

### Spectroscopy

An optical polarized‐light microscope (Zeiss AX10 Scope.A1) and spectrometer (Avantes Sensline AvaSpec‐HS2048) connected via a 600 µm diameter optical fiber were used in conjunction with a 20x objective (Zeiss EC Epiplan‐Apochromat 20 × 0.6 NA). Each sample was encapsulated between two glass slides within a 6 mm thick, 20 mm diameter rubber O‐ring and sealed with epoxy glue (Araldite Standard 2‐part epoxy resin). For Figure 3, three spectroscopic measurements were made at different locations across each sample and averaged, referenced against a silver mirror (Thorlabs PF10‐03‐P01 Ø1”). At each location, the spectrum was recorded independently through right and left‐circular polarized filters. For angle resolved spectroscopy, one measurement was taken for each sample using a laboratory‐built goniometer, as described by Chan et al.^[^ ^22^ ^]^ The same spectrometer and optical fiber as for the previous spectroscopic measurements were used, and the spectra referenced against a white diffuser (Labsphere SRS‐99‐010). The angle of incidence was fixed at 0° (normal to the sample surface) and the detector was rotated around the sample, allowing for the scattered light at increasing angles of reflectance to be quantified.

### Mechanochromicity

A new set of samples was prepared using the same sample formulation procedure described above. However, after centrifugation (33 °C, RCF 11,617 xg, 45 min) the formulations were poured into separate 1 cm thick, 5.5 cm diameter Petri dishes, sealed with parafilm and submerged as before into a water bath (33 °C) for a further 1.5–2 h. The Petri dishes were then stored at 4 °C for a minimum of 48 h, replicating the process as for all other samples. Given that HPC‐water can undergo inelastic lateral flow under compression, whereas HPC‐gel undergoes elastic deformation, the shape and size of the Petri dishes was fixed to constrain the geometry in terms of thickness and total volume. This allowed for the mechanochromic response of the two systems upon applying a compressive force to be directly compared. Mechanochromic data acquisition: A smartphone (Samsung Galaxy A5, rear‐facing camera) was used to acquire video footage of the sample during compression and release. A diffuse white light LED photography box was used as an illumination source and automatic camera settings used, except with the camera flash turned off and the focal length fixed. Samples were removed from the fridge and left to equilibrate to room temperature overnight. Before measurement, the Petri dish lids were removed and a thin polyvinyl chloride, PVC, film stretched over the exposed surface and subsequently cut to release any tension in the film that might influence the mechanochromic response. Samples were then left for a minimum of 15 min to ensure a rest color state. A pressure sensor (CSU15‐45N, SingleTact) was placed over the “press” location, video capture started, and a finger used to press into the sample surface, gently but firm, and released. Video data were captured for 25 s after removal of pressure and the material left at rest for a further 2 min to allow sufficient relaxation. The process was repeated to produce an averaged response. Mechanochromic analysis: Video data were recorded, and the ROI processed and analyzed using ImageJ (2.1.0/1.53c) and MATLAB_R2020a. Pixel RGB values from the ROI were converted into an average hue for each frame using the RGB to HSL color space transformation.^[^ ^18^ ^]^ By plotting the resultant average hue values as a function of time and normalizing between the pre‐compression baseline hue (the theoretical minimum) and the largest observed deviation in hue during/after compression (the theoretical maximum), the mechanochromic color relaxation response as a function of time in seconds was determined. The pressure sensor monitored the finger presses, and thus the force exerted, for each measurement to ensure an equal force was exerted onto each sample during different measurements, with the assumption that the finger exerts pressure over the entirety of the sensor's area. The variance in Figure 4c is thus attributed to any inhomogeneity in the force applied leading to a higher maximum force or a nonuniform mechanochromic response. The errors given for the linear mechanochromic response of the HPC‐gel are defined as the standard deviation from the trendline.

## Conflict of Interest

The authors declare no conflict of interest.

## Supporting information

Supporting Information

## Data Availability

Additional data relating to this publication are available from the University of Cambridge data repository (https://doi.org/10.17863/CAM.69768).
