# Supplementary material for: Mechanochromic, Structurally Colored, and Edible Hydrogels Prepared from Hydroxypropyl Cellulose and Gelatin
Source: Adv Mater. 2021 Jul 29;33(37):2102112. doi: 10.1002/adma.202102112 (PMC11468689; doi:10.1002/adma.202102112)
Supplement: Supplementary file 1 — Supporting Information [file ADMA-33-2102112-s001.pdf]

© 2021 Wiley-VCH GmbH

ADVANCED MATERIALS

Supporting Information

for *Adv. Mater.,* DOI: 10.1002/adma.202102112

Mechanochromic, Structurally Colored, and Edible Hydrogels Prepared from Hydroxypropyl Cellulose and Gelatin

*Charles H. Barty-King, Chun Lam Clement Chan, Richard M. Parker, Mélanie M. Bay, Roberto Vadrucci, Michael De Volder, and Silvia Vignolini**

Supporting Information

Mechanochromic, Structurally Colored and Edible Hydrogels prepared from Hydroxypropyl Cellulose and Gelatin

*Charles H. Barty-King, Chun Lam Clement Chan, Richard M. Parker, Mélanie M. Bay, Roberto Vadrucci, Michael De Volder and Silvia Vignolini**

C. H. Barty-King and Prof. M. De Volder
Department of Engineering, University of Cambridge, 17 Charles Babbage Road, Cambridge CB3 0FS (UK)

C. L. C. Chan, M. M. Bay, Dr. R. M. Parker, Dr. R. Vadrucci and Prof. S. Vignolini
Yusuf Hamied Department of Chemistry, University of Cambridge, Lensfield Road, Cambridge CB2 1EW (UK)

E-mail: sv319@cam.ac.uk

Keywords: hydroxypropyl cellulose, cholesteric liquid crystals, photonic hydrogels, mechanochromic materials, edible

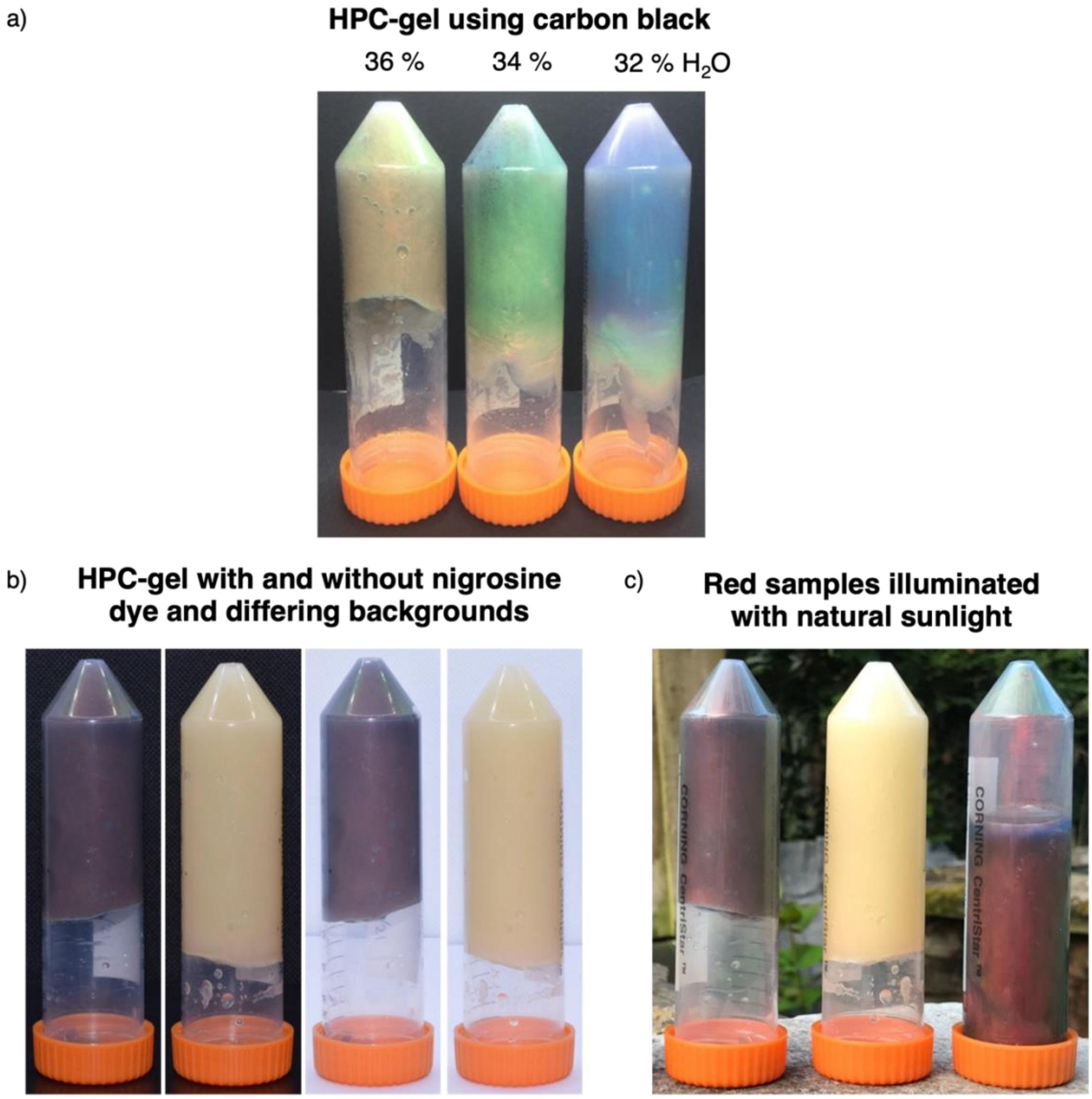

Figure S1. (a) Red, green and blue samples of HPC-gel, corresponding to 36, 34, 32 wt% water respectively, containing 0.05 wt% carbon black and sealed in 50 ml falcon tubes placed upside down for 48 h. **(b)** Red HPC-gel samples in-front of a black (*left*) and white (*right*) background after being placed upside down for 2 hours and photographed under direct illumination, with and without 0.005 wt% nigrosine dye. **(c)** Red HPC-gel with and without nigrosine dye (*left*), and HPC-water with nigrosine dye (*right*) after being placed upside down for 2 hours and photographed under natural sunlight.

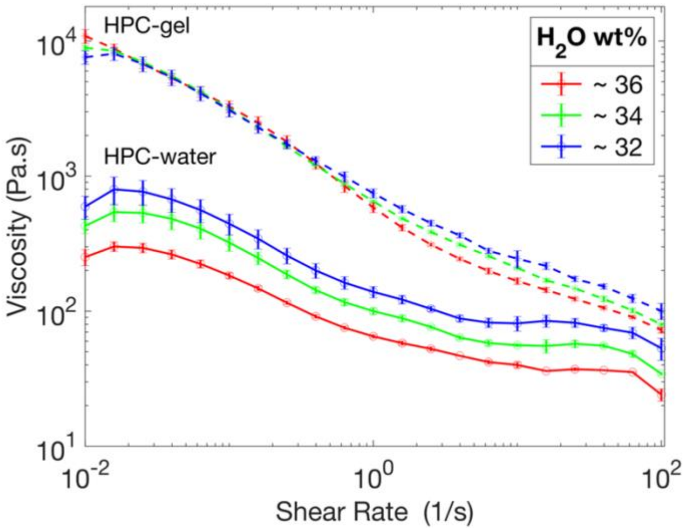

Figure S2. Viscosity-shear rate profiles where shear rate is ramped from 0.01 to 100 s^{-1} and the viscosity acquired. *Solid lines:* HPC-water. *Dashed lines:* HPC-gel. An average of three runs is shown for each plot, with standard deviation per point given in error bars. Pseudoplastic shear-thinning behaviour is observed in all samples, as well as an increase in viscosity with HPC concentration.

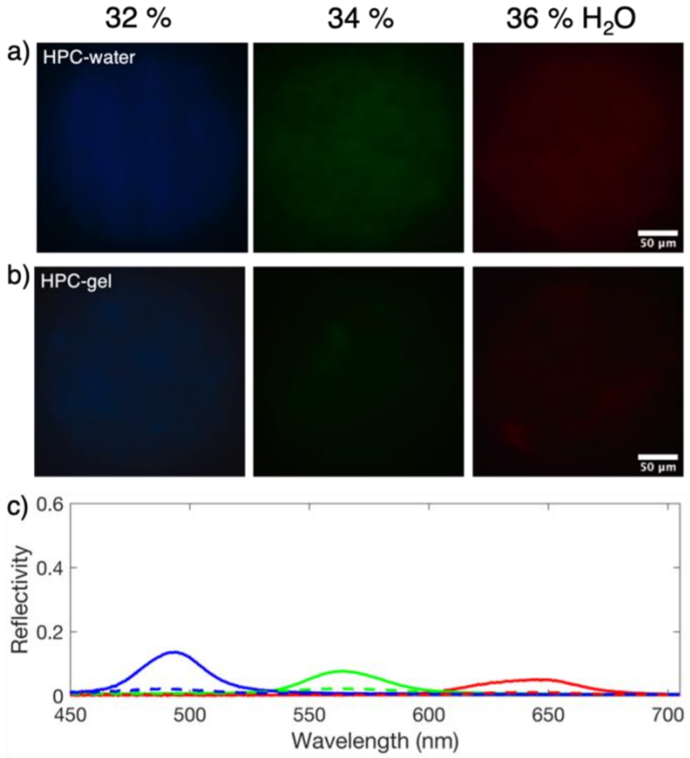

Figure S3. Brightfield microscope images comparing the optical appearances of **(a)** HPC-water and **(b)** HPC-gel for increasing water content (wt%) and imaged through a left-handed circular polarised filter. **(c)** Corresponding micro-spectroscopy of the HPC-water (*solid lines*) and HPC-gel (*dashed lines*). Each curve is an average of three spectra per sample and acquired at differing locations.

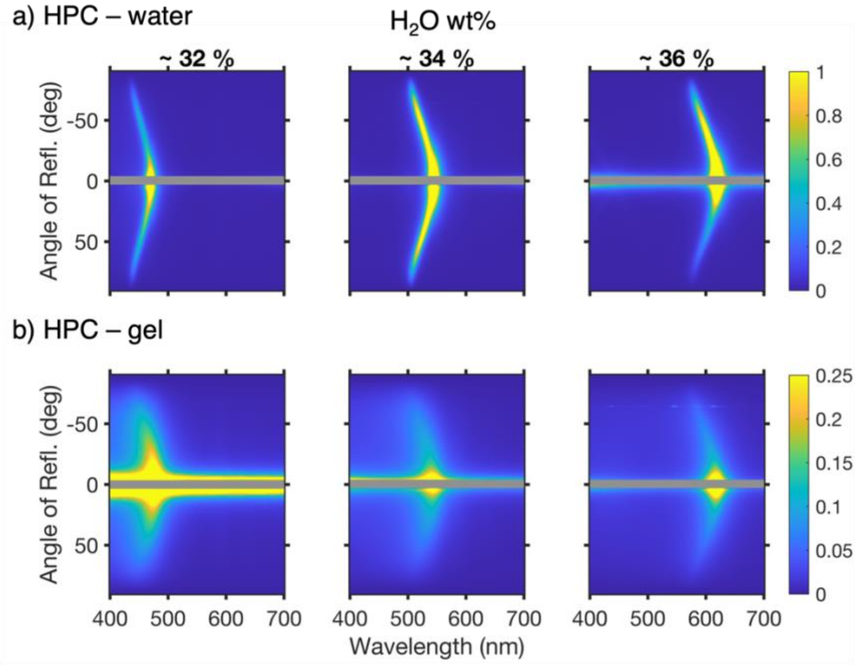

Figure S4. Heat-map goniometer plots of photonic HPC, captured at a fixed 0° angle of incidence. **a)** HPC-water; **b)** HPC-gel. Samples were rotated and the light scattered, with the angle of reflection (y) recorded against its wavelength (x). For ease of comparison, the HPC-gel plots have a different intensity scale (bar on right). The goniometer is mechanically unable to collect data between –14° to +14° and are therefore shaded out in grey.

S1. A basic description of the loss factor (rheology).

The loss factor, $tan\delta$, describes the ratio G″ : G′ where G″ and G′ respectively represent the viscous contributions (loss modulus) and elastic contributions (storage modulus).[1] Where $tan\delta > 1$, viscous contributions dominate and where $tan\delta < 1$, elastic contributions dominate, characterized respectively as liquid-like or viscoelastic solid-like (gel) behavior. When the storage and loss moduli contribute equally, a gel-transition point is reached and thus $tan\delta = 1$. The loss factor enables interpretations of the internal structure of a material, as well as its relaxation towards rest when the angular frequency is moving from high values (short term behaviour) to low values (long-term behavior towards rest).[1]

S2. RGB to HSL color space conversion for Figure 4.

Each pixel recorded by the smart phone camera is comprised of three primary color channels, red (R), green (G) and blue (B), mathematically represented as R = (255,0,0), G = (0,255,0) and

B = (0,0,255). For any given pixel, the relative weights of the R, G and B channels dictate its perceived color, for example, a reddish-orange may have the RGB coordinates (241,80,1). An RGB image is therefore comprised of an array of RGB coordinates, with each coordinate representing an individual pixel.[2] Subsequently, as RGB coordinates are vectoral by nature, they are challenging to plot on cartesian axes in an interpretable manner. A common color space conversion is used to resolve this difficulty, the RGB to HSL conversion, where H = hue, S = saturation and L = lightness (sometimes referred to as brightness). As only the color information, the hue, is of importance in this work, the S and L components are disregarded. Therefore, averaging all RGB coordinates from within the region of interest (ROI) provides a singular hue value for each frame of the video. To maintain consistent RGB pixel information density between frames and different samples, the magnification (zoom) of the camera and the total area of the ROI is fixed for all video acquisition and analysis steps.

For analysis of a video recording, a circular ROI is selected to be used over all frames. For a given frame, all pixels are averaged within the ROI to give an average RGB coordinate, converted into the HSL color space, S and L disregarded, and a singular average hue value obtained for each frame. Plotting the hue (y-axis) for each frame sequentially (x-axis) and using the framerate to calculate the time value for each frame, the change in color information is plotted through time. The frame rate is 30 fps. However, it is important to note that only the rate at which the hue returns to the pre-compression state is of interest to this work. Therefore, normalization of the hue values between the baseline for a given video - before a pressure is exerted (the theoretical minimum) - and largest deviation from that baseline (the theoretical maximum) i.e. the largest observed color change of the video being analyzed. Time zero is set at the frame of the theoretical maximum, being the normalized hue value of 1, and its recovery back towards the baseline observed as a function of time, as reported in Figure 4.

Assuming a first-order decay, the rate of recovery of the normalized hue represents the rate of the mechanochromic relaxation back to its initially colored state. From this, a time constant is calculated to represent the time taken for the color within the ROI to fall back to 1/e (≈ 36.8 %) of its initial value. A shorter time constant means a faster mechanochromic relaxation time; its 'refresh rate' if you will. This is because the color, and thus the internal photonic domains of the material, move back towards their rest state within a shorter time period.

The thicknesses of the HPC material also impacts the response time. However, by keeping a consistent thickness between all samples tested for mechanochromism, this variable is fixed. A consistent thickness was assured by casting the material into 1 cm tall petri dishes. Similarly, the container in which the HPC is cast hugely impacts the flow behaviour and deformation relative to a compression. However, by keeping the container consistent between tested samples, this variable is also fixed. The HPC-gel is free-standing, so could be tested for mechanochromism without a container, however for comparative purposes of this paper, no such experiments are presented.

S.3 Formulation challenges, and planetary centrifugal mixer tips and tricks

Three gelatin types (Sigma-Aldrich) were investigated: fish, bovine and porcine. These were chosen due to their low cost and ubiquity. Bovine gelatin worked comparably well to porcine gelatin in the HPC-gel system, but was prone to mould soon after formulation (within 5-10 days). Fish gelatin was unable to form solids (i.e. still flowed freely) in simple gelatin-water solutions, even after many days at rest and at very high gelatin contents > 60 wt%. Carrageenan was not used but could be tried as a 'vegetarian' alternative to animal-derived gelatin. The high bloom gelatin available from the supplier was tried in all cases.

Advice for homogeneous mixing in a planetary centrifugal mixing is given here for replicability purposes. During operation, vertical vortices induced within the container of a planetary centrifugal mixers container make it advisable to consider the order in which raw materials are added. Doing so facilitates homogeneous mixing of materials. Less dense materials should go in the container first, with incrementally denser material on top. For example, with HPC-gel, water is added first, followed by HPC and finally gelatin. Anecdotally, this order of addition produces the most homogeneous materials when using planetary centrifugal mixing. This was explained by the supplier as internal vortices during mixing lift materials from the middle of the container up and over towards the outsides of the container walls in a torus-configuration. By placing the least dense material in the container first, so they reside at the bottom, the least dense material is lifted up, through and over the denser material. In our HPC-gel example, the least dense material is water. Therefore, the aqueous component of the mixture is lifted up into, and then over, the denser HPC and gelatin material above. This is efficient for homogenous mixing as to flip the ordering means denser material reside at the bottom of the container and require more energy, and thus more aggressive mixing parameters, to lift up, through and over the less dense material above. These vortices also mean a high height-to-diameter aspect ratio of the chosen mixing container usually give better mixing; a column shaped container is preferable to a disk-shaped container. The high aspect ratio causes the material being lifted in a torus-configuration to travel up through column over longer distances and thus increases interaction between the material compared to lower aspect ratio containers of the same volume.

References

[1] T. G. Mezger, *Applied Rheology – With Joe Flow on Rheology Road*, Anton Paar Ltd., **2015**.

[2] H.-L. Liang, M. M. Bay, R. Vadrucci, C. H. Barty-King, J. Peng, J. J. Baumberg, M. F. L. De Volder, S. Vignolini, *Nature Communications* **2018**, *9*, 4632.
